# Interprofessional clinical training in mental health improves students’ readiness for interprofessional collaboration: a non-randomized intervention study

**DOI:** 10.1186/s12909-019-1465-6

**Published:** 2019-01-18

**Authors:** Michael Marcussen, Birgitte Nørgaard, Karen Borgnakke, Sidse Arnfred

**Affiliations:** 1 Department of Clinical Medicine, University of Copenhagen and Psychiatry Slagelse, Region Zealand, Fælledvej 6, 4200 Slagelse, Denmark; 2 Department of Public Health, University of Southern Denmark, Copenhagen, Denmark; 3 Department of Media, Cognition and Communication, University of Copenhagen, Copenhagen, Denmark

**Keywords:** Interprofessional education, IPE, Team collaboration, Psychiatry

## Abstract

**Background:**

Over the past decades, the health sector in general has increasingly acknowledged the effectiveness of interprofessional clinical training in enhancing teamwork. In psychiatry, however, knowledge of the benefits of collaborative clinical training is sparse. This study aimed to investigate the impact of interprofessional training on students’ readiness for interprofessional collaboration in a psychiatric ward.

**Methods:**

An intervention study assessed interprofessional clinical training in a training ward. Undergraduate students from the disciplines of medicine, nursing, psychotherapy, pedagogy, and social work were allocated either to an intervention group receiving interprofessional training or to a comparison group receiving conventional clinical training. Outcomes were assessed using the Readiness for Interprofessional Learning Scale (RIPLS) and the Assessment of Interprofessional Team Collaboration Scale (AITCS). Linear mixed regression was used to compare differences in mean scores postintervention, adjusted for baseline score, gender, and profession.

**Results:**

Mean postintervention scores were higher in the intervention group (*n* = 87) than in the comparison group (*n* = 108) for both scales (overall sum score). For the RIPLS, the mean difference was 2.99 (95% CI 0.82 to 5.16; *p* = 0.007); for the AITCS it was 8.11 (95% CI 2.92–13.30; *p* = 0.002). Improvement in readiness for interprofessional learning and team collaboration in the intervention group remained statistically significant after adjustment for baseline differences between the two groups.

**Conclusion:**

Students’ self-reported readiness for interprofessional learning and their team collaboration were improved after interprofessional clinical training. Still, further studies of both the processes and the long-term effects of undergraduate IPE in mental healthcare are needed. The study was registered March 62,017 on ClinicalTrials.gov: NCT03070977 (Retrospectively registrered).

**Electronic supplementary material:**

The online version of this article (10.1186/s12909-019-1465-6) contains supplementary material, which is available to authorized users.

## Background

Many professionals working in mental healthcare have insufficient skills to participate effectively in interprofessional teamwork [1], and collaboration among team members continues to pose a challenge [1–4]. This might be due to professional cultures and poor communication [1, 2], which has consequences for the quality of care (i.e. errors and poor service delivery). For that reason, collaborative practices are increasingly being introduced in wards, as they have shown their effectiveness for mentally ill people with complex needs [5–7]. Interprofessional education (IPE) continues to be proposed by policymakers in mental healthcare as a means of improving collaborative practices and patient care, i.e. teamwork is invoked as by policymakers as effective to prevent relapse and manage chronic conditions [5, 6]. IPE is assumed to be effective to enhance collaborative practice despite challenges to demonstrate its effectiveness in clinical education in mental health [2, 4, 8, 9]. The World Health Organization (WHO) defines IPE as settings in which “(…) students from two or more professions learn about, from and with each other to enable effective collaboration and improve health outcomes” [6]. However, previous studies have reported challenges attached to IPE, such as logistical barriers to plan IPE sessions, when students come from different programs and universities. Moreover, IPE sessions is challenged by different academic levels of the students and prior experiences to interprofessional collaboration [8, 9]. Acknowledging the difficulties involved in achieving interprofessional collaboration, WHO recommends that the fostering of IPE begin at undergraduate level [6]. Various IPE initiatives for undergraduates have been described, some of which have taken place in clinical settings where students from different healthcare professions work together [10–13]. Hospitals are increasingly establishing such clinical training wards [10, 12, 13] but, to date, only initiatives in other specialties have been described [10, 11, 14–16]. Interprofessional clinical training aims to improve students’ attitudes toward and awareness of other professions and their roles, and to advance teamwork and collaborative competencies. However, demonstrating change in interprofessional attitudes and behavior remains difficult and, in some settings—such as mental health education—IPE remains underresearched [1, 17]. Those studies that have been undertaken in mental health education contexts have produced limited results. Marcussen et al.’s (2018) systematic review of the effects of undergraduate IPE in mental health practice found only eight studies qualifying for inclusion [17]. This calls for intervention studies with more rigorous research designs and methods [1, 17] . Therefore, this study contributes to the sparse literature in the field of undergraduate IPE in clinical mental health. Additionally, Anderson et al. [18] proposed the role of questionnaires as establishing a baseline for regular assessment of change. We used a similar approach. Moreover, given the positive results gained in other specialties, we set out to investigate whether similar results could also be achieved in psychiatric wards.

Thus, we investigated the impact of interprofessional training on students’ readiness for interprofessional collaboration in a psychiatric ward. The study took place during 2016–2018 in the Department of Adult Psychiatry in Slagelse, Denmark. The department has four inpatient wards, an outpatient clinic, and an emergency ward, serving a mixed urban and rural district.

An interprofessional training ward was established in the department for students of medicine, nursing, pedagogy, physiotherapy, and social work. The organization of the clinical training ward was inspired by the work of Nørgaard et al. [19] and aimed to create a new environment for learning, to enable students learn from each other and develop their competences in interprofessional collaboration and reflection [20]. Reflection is considered a key strategy in interprofessional training [21, 22]. The intervention was studied in a prospective clinical trial, which enabled a robust comparison between those who had been exposed to the intervention and the comparison group. This paper describes the assessment and its results.

## Methods

### Design

**Fig. 1:**
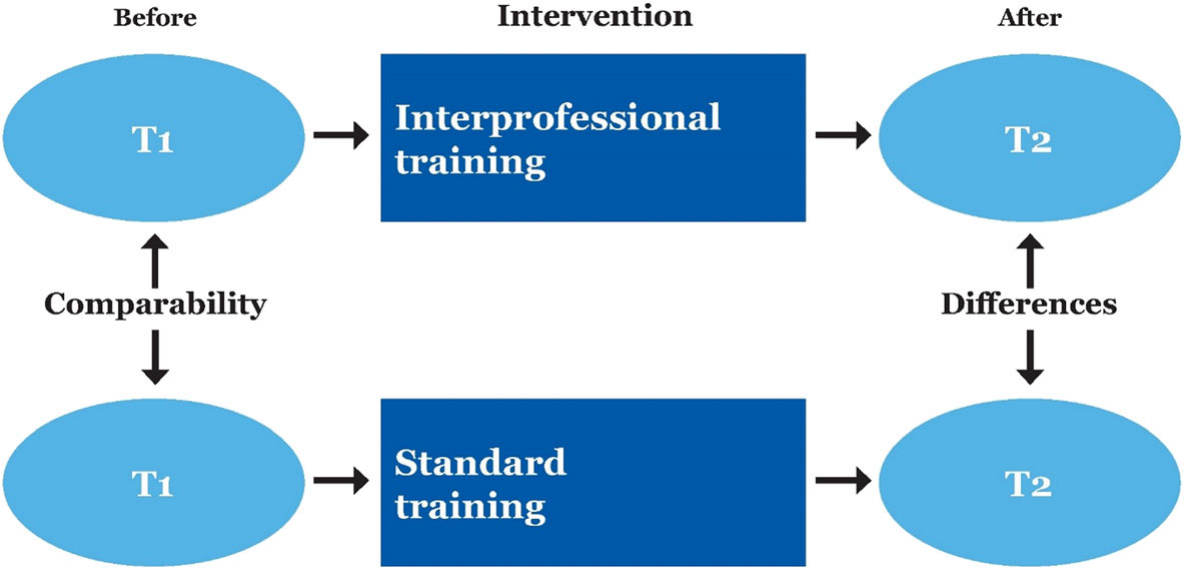
Study design

We designed a prospective clinical trial with a comparison group, as shown in Fig. 1. The intervention followed the time of clinical placement. The students in the intervention group received interprofessional clinical training, while those in the comparison group received conventional clinical training. Data collection took place from October 2016 to March 2018. Self-report questionnaires were administered to both groups before and after clinical training. The design allowed us to determine the changes that occurred (by comparing the scores after clinical training (T2) with those from the baseline (T1)) [20]. The study was registered on ClinicalTrials.gov (NCT03070977), and conducted in accordance with the CONSORT guidelines [23].

### Participants and setting

The study participants were students on clinical placements in psychiatric inpatient wards. The patients, who were aged 18 years and above, suffered from psychiatric disorders such as schizophrenia, psychosis, major depression, bipolar disorder, or severe personality disorder.

The 195 students who were eligible for participation were studying medicine (in the 5th year), nursing (2nd and 3rd years), physiotherapy (3rd year), social and healthcare (2nd year), pedagogy (3rd year), and social work (2nd year). Furthermore, the learning outcomes were discipline specific, however, the self-reported outcomes in this study were the same for both groups. Although the timing of interprofessional clinical training differed for the various professions, the students were assumed to have reached a stage in their education at which they had developed a professional identity and were capable of contributing to interprofessional learning.

The students from each of the professions were evenly allocated to the intervention and the comparison group by course administrators from their universities and colleges, with no involvement of the research team.

### Intervention

In 2015, an interprofessional study unit was established with 17-beds. This psychiatric ward was organized in 3 clinical care teams including professionals from medicine, nursing, psychology, pedagogue, social work, and the patient, as well as students. A facilitator team was responsible for the interprofessional training. In medio 2016 the facilitation team completed a course facilitating interprofessional collaboration and training. The intervention involved the total staff participation in an initial workshop. The intervention consisted of two types of activities: clinical care teamwork (mainly supervised by instructors from each of the participating professions) and interprofessional group tutorial sessions led by instructors with extensive experience in delivering IPE. In addition to strengthening students’ own professional roles, the training aimed to increase their knowledge of other professionals’ roles and to develop interprofessional collaboration.

The students participated in a workshop with group discussions, small-group work, and PowerPoint presentations introducing the primary diagnoses found in the ward and the responsibilities of each of the health professions involved in care. These scenarios provided a basis for instructor-facilitated discussions related to living with a psychiatric disorder, understanding the patients and their backgrounds, and the roles of healthcare teams.

### Interprofessional group turorial

To improve their reflection on clinical practice [2] and to strengthen their knowledge of the patients’ treatment and care, all students met once a week for interprofessional group tutorial. Besides the IPE facilitation team responsible for planning the group tutorial, a nurse facilitator was present during all sessions.

The group tutorial required the students to prepare and deliver a presentation to their peers and instructors. The patient’s view of the condition, and how it was managed in the ward, were obligatory elements of the presentations, as was the presenter’s suggestions for improvements facilitated through interprofessional collaboration. In subsequent group discussions facilitated by the tutors, interprofessional practices and challenges in the care of patients with complex needs were highlighted. Every session was structured on real patients, i.e.: a patient suffering from severe mental disorders such as schizophrenia and co-occurring substance use disorders traditionally received treatments for their two disorders from two different sets of clinicians in parallel treatment systems.

### Clinical care teams

In the training ward, the students’ clinical training was organized in care teams of between three and ten students [7, 13] alongside with the professionals. Weekly conferences were held with each team to ensure that the care and treatment were well planned and well coordinated. To further support this aim, the conferences were attended by permanent staff, the patient, and his or her family during hospitalization. The students were distributed between the care teams for supervision, which took place in collaboration with the IPE facilitators.

### Comparison group

Students in the comparison group continued their usual discipline-specific training during their clinical placement, with no structured interprofessional training. Their clinical training was organized in a uniprofessional structure, as opposed to the team-organized structure used in the intervention group.

### Outcome measures

Outcomes in both groups were measured at the beginning (T1) and at the end of the clinical placement (T2). The time between T1 and T2 varied due to differences in the duration of clinical training between professions (3–12 weeks).

#### Readiness for Interprofessional learning

Self-reported readiness for interprofessional learning were measured using the scale with the same name (RIPLS), as modified by McFadyen et al. [24], which has been found to have good internal consistency [25–27]. The Danish four-subscale version with 29 items applied here has been validated and culturally adapted by Nørgaard et al. [27]. The learning scale assesses *Teamwork and Collaboration*, *Negative Professional identity*, *Positive Professional identity*, and *Roles and Responsibilities* [28] using a five-point Likert scale: (Strongly disagree (1), Disagree (2), Undecided (3), Agree (4), Strongly agree (5)).

#### Team collaboration

To gain insight into the students’ self-reported level of collaboration, we used the Assessment of Interprofessional Team Collaboration Scale (AITCS) of Orchard et al., with 47 items in four subscales (Partnership, Cooperation, Coordination, and Shared decision making), using a 5-point Likert scale [29]: (Never (1), Rarely (2), Occasionally (3), Most of the time (4), Always (5)). We employed the Danish version validated by Hellman et al. [30], with 37 items distributed on three subscales (Partnership/Shared decision making, Cooperation, and Coordination).

### Ethical considerations

The students were informed of the project and its purpose immediately before participation. Responding to the questionnaire was considered to constitute voluntary consent to participation. Data were entered into the EasyTrial online Clinical Trial Management system. Data were labelled with unique identifiers, and all personal identifiers were removed or disguised during analysis to preclude personal identification. Raw data are available as supplementary files (Additional files 1 and 2). The study was approved by the Danish Data Protection Agency (2008-58-0020), and thus required no further ethical approval, according to Danish legislation (16–000014).

### Analysis

The sample size was calculated on the basis of a type I error (α) of 5% and a type II error (β) of 20% (with a power of 80%). We used a standard deviation of eight points, based on findings from an intervention study [31] using the RIPLS (our primary outcome). Anticipating a withdrawal rate of 20%, we allocated 90 participants to each group.

The participants were described in terms of gender and profession. To investigate subscale assumptions, we conducted a confirmatory factor analysis at baseline on the full dataset of students. Internal consistency between items was assessed using Cronbach’s alpha. Both scales (RIPLS and AITCS) use a Likert scale. The scale scores were treated as interval as recommended in previous studies [32, 33]. We initially used unpaired *t*-tests to assess mean score differences at baseline. Differences over time were explored using the paired sample *t*-test. The postintervention mean scale scores were compared between groups and over time using linear mixed regression, and adjusted for gender, profession, and baseline scores. The Bonferroni test was used to correct the significance level when multiple comparisons were made. All statistical analyses were performed using SPSS (IBM Corp. Released 2018. IBM SPSS Statistics for Windows, Version 24.0. Armonk, NY: IBM Corp).

## Results

### Population

**Fig. 2:**
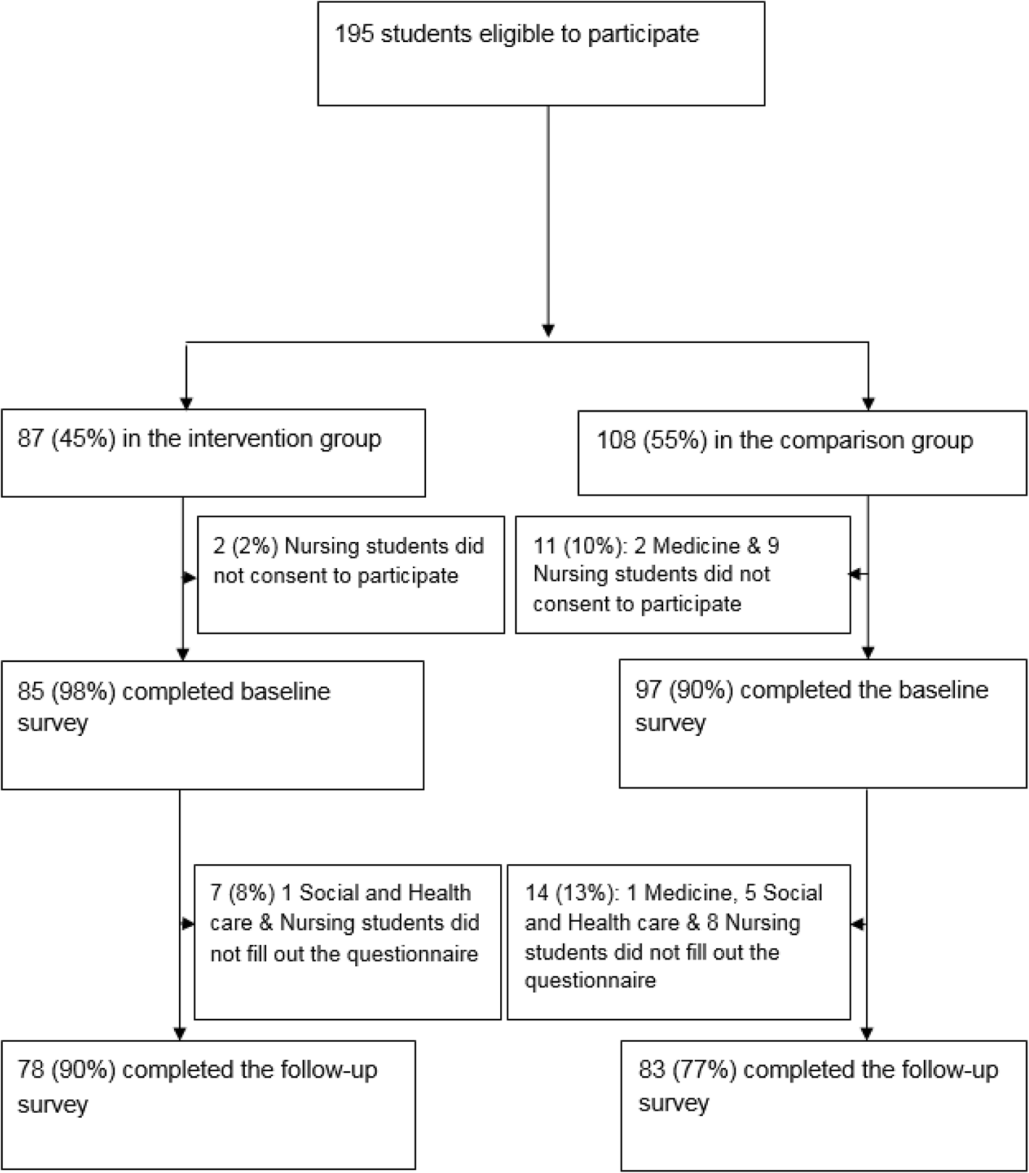
Flow chart of study participants

In Fig. 2, we present the flow of participants through the study. A total of 87 students were allocated to the intervention group, with 108 in the comparison group. Baseline and postintervention data were collected for 78/87 intervention group participants (98%) and 83/108 for the comparison group participants (77%), respectively. As Table 1 shows, baseline characteristics were comparable between the intervention and the comparison participants.

**Table 1:** Gender and profession of responding students

	Respondents					
	T1			T2		
	Intervention (*n* = 85) n (%)	Comparison (*n* = 97) n (%)	P^a^	Intervention (*n* = 78) n (%)	Comparison (*n* = 83) n (%)	P^a^
*Gender*						
Male	14 (16.5)	12 (12.4)	0.4	14 (18.0)	11 (13.3)	0.4
Female	71 (83.5)	85 (87.6)		66 (82.1)	72 (86.8)	
*Profession/Study*						
Nurse	44 (51.8)	51 (52.6)	0.09	38 (48.7)	43 (51.8)	0.1
Medical	14 (16.5)	17 (17.5)		14 (18.0)	16 (19.3)	
Social and healthcare	20 (23.5)	29 (29.9)		19 (24.4)	24 (28.9)	
Other^b^	7 (8.2)	0 (0.0)		7 (9.0)	0 (0.0)	

*T1* Time 1 (baseline) measurement, *T2* Time 2 measurement (after clinical training). Social and healthcare = nursing assistant

^a^Chi-square test

^b^Other = Students from Pedagogy, Physiotherapy, and Social work

On both scales, the baseline scores for the intervention group and the comparison group were comparable, as evident from the estimates and *p*-values (Table 2).

**Table 2:** Crude scores at baseline and postintervention

	Respondents					
	T1^a^			T2^b^		
	Intervention (n = 78) (mean; SD)	Comparison (*n* = 83) (mean; SD)	P	Intervention (*n* = 78) (mean; SD)	Comparison (*n* = 83) (mean; SD)	P
*Total RIPLS score*	114.37; 8.92	115.02; 9.16	0.7	117.39: 6.57	114.80; 9.78	0.001
*RIPLS subscales*						
Teamwork and collaboration	37.71; 4.87	38.31; 3.95	0.4	39.95; 3.43	38.43: 3.96	0.001
Negative professional identity	12.51; 2.22	12.95; 2.08	0.2	12.94; 1.90	12.75; 2.20	0.05
Positive professional identity	16.80; 2.24	16.84; 2.41	0.9	17.23; 2.12	16.43; 2.54	0.05
Roles and responsibilities	47.36; 4.48	46.92; 4.52	0.5	47.27; 4.47	47.18; 4.56	0.8
	Intervention (*n* = 69) (mean; SD)	Comparison (*n* = 79) (mean; SD)	P	Intervention (*n* = 69) (mean; SD)	Comparison (*n* = 79) (mean; SD)	P
*Total AITCS score*	154.80; 20.99	153.49; 18.98	0.7	162.33; 19.24	153.27; 20.81	0.001
*AITCS subscales*						
Partnership/shared decision making	80.02; 10.91	79.34; 9.84	0.7	83.54; 9.43	78.77; 10.68	0.001
Cooperation	46.67; 6.85	45.86; 6.38	0.5	49.07; 6.50	46.23; 7.32	0.001
Coordination	28.12; 5.28	28.29; 4.18	0.8	29.73; 4.60	28.27; 4.31	0.003

*RIPLS* Readiness for Interprofessional Learning Scale, *SD* Standard deviation, *AITCS* Assessment of Interprofessional Team Collaboration Scale

^a^Independent Samples *t*-test

^b^Paired *t*-test

Both scales were tested for internal reliability, and Cronbach’s alpha was estimated at 0.77 (RIPLS) and 0.97 (AITCS) in overall reliability, which is considered acceptable [27].

### Effect of interprofessional training

The mean scores (overall summed scores) on both scales increased for the students in the intervention group after completion of the interprofessional clinical training. As seen in Table 2, the pre- and post total RIPLS scores were 114.37–117.39 in the intervention group and 115.02–114.80 in the comparison group. This improvement for the intervention group was statistically significant (*p* = 0.001). Similarly, the pre- and post total AITCS scores were 154.80–162.33 in the intervention group and 153.49–153.27 in the comparison group, respectively. This improvement for the intervention group was also significant (*p* = 0.001).

Table 3 presents the differences in the mean scores over time for both groups, and for both scales. The mean postintervention scores on a five-point scale, adjusted for gender, profession, and baseline, were higher in the intervention group than in the comparison group. Linear mixed regression was used to adjust for baseline characteristics (Table 3). The mean difference between the two groups was: 2.99 (95% CI 0.82 to 5.16; *p* = 0.007) in total RIPLS score with an effect size, Cohen’s d of 0.4. The improvement in the total score for the intervention group was statistically significant for the subscales as well: 1.76 (95% CI 0.74 to 2.78; *p* = 0.001) regarding teamwork and collaboration, plus 0.86 (95% CI 0.32 to 1.40; *p* = 0.002) for positive professional identity. No significant differences were found between the two groups in terms of negative professional identity, and roles and responsibilities. The mean difference in total AITCS score was 8.11 (95% CI 2.92–13.30; *p* = 0.002) with an effect size, Cohen’s d of 0.5, which is considered as a moderate effect [34, 35]. For partnership and shared decision-making, this was 4.26 (95% CI 1.63–6.90; *p* = 0.002); for cooperation, 2.30 (95% CI 0.49–4.10; *p* = 0.01); for coordination, 1.55 (95% CI 0.37–2.72; *p* = 0.01). Both scales showed that the mean score difference in the intervention group was larger than that of the comparison group, although adjustment (by gender, age and professions) minimized the difference (Table 3).

**Table 3:** Estimates of RIPLS and AITCS over time and between groups ^a b^

	Intervention (*n* = 78) (mean; SE)	Comparison (*n* = 83) (mean; SE)	Between group (mean diff.)	CI (Difference-CI)	P
Total RIPLS score	117.59; 0.65	114.60; 0.76	2.99	0.82–5.16	0.007
Subscales					
Teamwork	40.07; 0.37	38.32; 0.36	1.76	0.74–2.78	0.001
Negative professional Identity	13.09; 0.18	12.61; 0.17	0.48	−0.01–0.97	0.05
Positive professional Identity	17.26; 0.20	16.41; 0.19	0.86	0.32–1.40	0.002
Roles and responsibilities	47.11; 0.38	47.33; 0.37	−0.23	−1.28–0.83	0.7
	Intervention (*n* = 69) (mean; SE)	Comparison (*n* = 79) (mean; SE)	Between group (mean diff.)	CI (Difference-CI)	P
Total AITCS score	161.82; 1.92	153.71; 1.79	8.11	2.92–13.30	0.002
Subscales					
Partnership/decision making	83.27; 0.97	79.01; 0.91	4.26	1.63–6.90	0.002
Cooperation	48.78; 0.67	46.48; 0.62	2.30	0.49–4.10	0.01
Coordination	29.78; 0.43	28.23; 0.41	1.55	0.37–2.72	0.01

*IPE* Interprofessional clinical training, *Standard* Standard clinical training (Comparison group), *RIPLS* Readiness for Interprofessional Learning Scale, *SE* Standard error, *AITCS* Assessment of Interprofessional Team Collaboration Scale

^a^Linear mixed regression (with Bonferroni correction)

^b^Mean scores in the comparison and intervention groups were adjusted for baseline scores, profession, and gender

## Discussion

This intervention study has found that interprofessional clinical training yield a moderate improvement in students’ self-reported readiness for interprofessional learning. Likewise, moderate improvements were also found in the intervention group in terms of students’ self-reported team collaboration, as compared to the comparison group. Consistent results were found using both the RIPLS scale and the AITCS scale. We likewise found significant improvements in the professional identity of the intervention group students as team members. In terms of roles and responsibilities, no significant differences were identified between the groups— a lack of difference for which no clear explanation emerged. However, Barnett et al. found a similar result in their intervention study of interprofessional attitudes arising from shared learning in mental health (36)]. Likewise, the systematic review of Marcussen et al. [17] concluded that students of mental health responded well to IPE, especially in terms of improved collaboration skills and more positive views of other professions’ contributions.

The significantly improved RIPLS and AITCS scores found after interprofessional training in our study corroborates the findings of previous studies showing that interventions using active training methods lead to higher RIPLS scores and larger difference in mean score than interventions using passive methods [37]. Our study’s use of active training methods seems to have had a moderate effect. Similarly, Pollard et al. [38] found that students value practical interprofessional experience over what can be gained in simulated environments in university settings. Moreover, they seem to appreciate training these skills alongside experienced professionals, and with real patients in real contexts [38]. Reeves and Pauzé [1, 4] have emphasized undergraduate learners’ benefit from the inclusion of real-world thinking in IPE.

With regard to the impact of IPE in mental health education, the quality of evidence is critical to advancing our understanding of ways of improving IPE. Studies undertaken in mental health contexts have so far produced limited or disappointing results. Barnett’s study (36)] conducted in a youth mental health service has shown a strengthening of students’ positive attitudes toward other professions and interprofessional learning following participation in an IPE workshop. An IPE study in mental health by Priest et al. [8] has clarified roles and practical collaboration, although no change in professional identity was found. Many challenges were identified by Priest et al. [8], including some that arose from differences in academic levels among student groups, their previous experience, and assessment. Studying an in-service IPE initiative among community mental health teams, Reeves and Freeth [2] showed that while the educational input was well received, wider success was elusive, as already agreed plans for collaboration were not implemented. Our systematic review of the effects of interprofessional education in mental health practice found only eight studies qualifying for inclusion [17]. This calls for more rigorous research designs and methods [1, 4, 17]. However, despite these challenges, it remains important to offer collaboration with future colleagues as a foundation for effective teamwork in mental health treatment and care.

The baseline RIPLS scores for the participants in this study were almost identical to those found in a large Australian sample of undergraduate paramedic and nursing students [39] and in a UK sample of nursing and psychology students [8]. Both studies found benefits related to IPE; however, investigation of the effect of IPE in relation to more than two disciplines has been sparse and previous results may thus not be comparable with those published here.

Both Wakely et al. [40] and Wellmon et al. [41] found improvement after interprofessional clinical training similar to our findings: The pre- and post RIPLS scores for the subscale: team work and collaboration were in Wakely’s study 38.5–41.0 and in Wellmon’s study 37.91–39.91, respectively. As seen in Table 2 the pre- and post RIPLS scores in our study were 37.71–39.95 in the intervention group.

Also, when using the AITCS scale, we found statistically significant improvements in teamwork in the intervention group. We can thus corroborate the results of a Canadian study [42] that used the AITCS survey to investigate attitudes of interprofessional collaboration in an IPE unit (a 30-bed inpatient medical unit). However, our results and the Canadian results also indicate that many areas of teamwork still need to be addressed, such as the need for organizational support for teamwork. So far, only a few studies have substantiated the positive effects of educational outcomes within mental health educational research [1, 17]. This may be due to a number of different factors, such as the challenges inherent in the measurement of outcome assessments, different objectives within teams, and differences in local contexts [2, 8].

### Strengths and limitations

The interprofessional training approach was a strength of this study, which appears to have supported the students in gaining interprofessional skills. The experimental design enabled robust analysis of changes in students’ attitudes in both the intervention group and comparison group. The lower response rate in the comparison group (77%) than in the intervention group (90%) should be noted, as it may have weakened the generalizability of these findings. It is reassuring, however, that the response rates were similar across the study groups (i.e., the future professions). Although allocation to intervention or comparison group was not random, the allocation procedure was completely independent of the study hypothesis. This is similar to the approach taken in previous IPE studies [43, 44]. No baseline differences were identified between intervention and comparison groups. Both the scales (RIPLS and AITCS) used here to measure readiness for interprofessional learning and team collaboration have previously been validated for use with healthcare students [27, 29, 30], and thus allow for comparison with similar studies. We identified weak internal consistency in the RIPLS subscale: Roles and Responsibilities (with a Cronbach’s alpha = 0.59). This is also reported in previous studies [26, 27, 45]. Although the difference in the length of the clinical training periods of the student groups is a limitation, it is one that our study shares with several previous IPE studies [7–9, 36, 46]. The findings are based on self-reported measures of readiness for interprofessional learning and team collaboration. Completion of the surveys was voluntary, and it is possible that the students self-reported views may not be representative of all participants in the inclusion period.

### Implications for mental health education

The findings of this study indicate that training in interprofessional wards can meet its objectives, and that interprofessional clinical training can be effective in fostering positive attitudes toward working with other professions. Furthermore, it is possible that embedding IPE learning within the context of hospital wards and using a team-based approach may enhance students’ learning and their ability to translate IPE principles into “real-world” thinking. These tentative explanations require further exploration in studies using qualitative methodologies, as well as in prospective, long-term studies [47]. Process evaluations of future IPE interventions may help clarify which elements have the strongest impact on learning outcomes, so that these can be optimized, as suggested by our systematic review of IPE in mental health to undergraduates [17]. In considering the outcomes of this study, it may be relevant to stress the benefits of having an established teaching team with excellent skills in facilitating the integration of students in interprofessional settings.

## Conclusion

Students’ self-ported readiness for interprofessional learning and their team collaboration were improved after interprofessional clinical training, as compared with clinical training as usual. The present study contributes key information to the planning of interventions in organizational settings. Nevertheless, further studies of both the processes and the long-term effects of undergraduate IPE in mental healthcare are needed.

## Additional files

Additional file 1: RIPLS Spreadsheet. Readiness for Interprofessional Learning Scale (RIPLS) dataset. (XLSX 57 kb)

Additional file 2: AITCS Spreadsheet. Assessment of Interprofessional Team Collaboration Scale (AITCS) dataset. (XLSX 46 kb)

## Abbreviations

AITCS: Assessment of Interprofessional Team Collaboration Scale
IPE: Interprofessional education
RIPLS: Readiness for Interprofessional Learning Scale
